# Secular Trends of Underweight, Overweight, and Obesity in Children and Adolescents from Ukraine

**DOI:** 10.3390/ijerph18063302

**Published:** 2021-03-23

**Authors:** Katarzyna Dereń, Justyna Wyszyńska, Serhiy Nyankovskyy, Olena Nyankovska, Marta Yatsula, Edyta Łuszczki, Marek Sobolewski, Artur Mazur

**Affiliations:** 1 Institute of Health Sciences, Medical College of Rzeszow University, 35-959 Rzeszow, Poland; justyna.wyszynska@onet.pl (J.W.); nianksl@gmail.com (S.N.); eluszczki@ur.edu.pl (E.Ł.); 2 Pediatrics Department #1, Danylo Halytsky L’viv National Medical University, 79010 Lviv, Ukraine; dr.yatsula@gmail.com; 3 Department of Pediatrics and Neonatology, Danylo Halytsky L’viv National Medical University, 79010 Lviv, Ukraine; lena.nyank@gmail.com; 4 Faculty of Management, Rzeszow University of Technology, 35-959 Rzeszow, Poland; mareksobol@poczta.onet.pl; 5 Institute of Medical Sciences, Medical College of Rzeszow University, 35-959 Rzeszow, Poland; drmazur@poczta.onet.pl

**Keywords:** obesity, children, underweight, overweight

## Abstract

Overweight and obesity, as well as underweight in children and adolescents, pose a significant public health issue. This study aimed to investigate the secular trend of the incidence of underweight, overweight, and obesity in children from Ukraine in 2013/2014 and 2018/2019. The studies were conducted in randomly selected primary and secondary schools in Ukraine. In total, 13,447 children (6468 boys and 6979 girls) participated in the study in 2013/2014 and 18,144 children (8717 boys and 9427 girls) participated in 2018/2019. Measurements of body weight and height were performed in triplicate. Underweight, overweight, and obesity were diagnosed according to the standards of the World Health Organization (WHO). In the group of girls, a significant difference between 2013/2014 and 2018/2019 measurements was found only among 7-year-olds. The percentage of girls at this age exceeding the body mass index (BMI) norm was lower in the 2018/2019 study. In boys, a significant difference was also found in 7-year-olds, and, as in girls, a lower share of overweight and obesity was found in 2018/2019. But for the ages of 12, 13, and 15, the significant differences had a different character—more overweight or obese boys were found in the 2018/2019 study. The proportion of underweight children was similar for the majority of age groups in both genders and did not differ in a statistically significant way.

## 1. Introduction

Childhood obesity is a major public health problem. The prevalence of obesity among children is increasing and may negatively affect their immediate health [1,2]. The problem of being underweight affects countries with different socioeconomic statuses, and is more common among young women, especially in Europe [3]. Low body mass index (BMI) for age can have serious consequences for a child’s development, health, and well-being. It may reflect malnutrition and result from poor nutritional practices or various medical conditions [4]. On the other hand, obesity in children and adolescents significantly increases the risk of type 2 diabetes, hypertension, and cardiovascular and liver conditions, which are likely to persist into adulthood, leading to long-term health impairment and increased morbidity and mortality in adults [5]. In addition to the immediate and long-term adverse health consequences, childhood overweight and obesity have a negative impact on children’s self-esteem, confidence, and academic performance [6].

Obesity is increasing worldwide, affecting children and adults in both developed and developing countries [7,8]. Over 340 million children and adolescents aged 5–19 were overweight or obese in 2016 [9]. Between 1975 and 2016, the literature shows that the mean BMI of boys and girls aged 5–19 years around the world increased from 16.8 to 17.2 kg/m^2^, respectively [10]. In contrast, the BMI trend in 2020 in the world has shown that the BMI trend of school children and adolescents varies across countries and territories. These country differences show that childhood and adolescence are the key periods in terms of BMI change and its lifelong health impact. The BMI of children and adolescents is highly variable across countries, which also indicates heterogeneous nutritional quality [11]. Particularly noteworthy is the growth rate of overweight and obesity among children and adolescents. Measuring the prevalence of childhood obesity and monitoring changes over time is important for population health surveillance and can facilitate the design of prophylaxis and treatment interventions. A number of socio-demographic factors are also associated with the increased prevalence of overweight and obesity [12]. Given the rapidly increasing morbidity and health risk, obesity in children and adolescents requires continuous monitoring [5].

Until now, no studies have analyzed changes in the prevalence of overweight and obesity as well as weight deficiency in children in Ukraine. This study assessed the prevalence of underweight, overweight, and obesity among children and adolescents in a representative sample by using BMI, according to international definitions, and the short-term changes that have occurred in the prevalence of underweight, overweight, and obesity in subjects of this age.

## 2. Materials and Methods

This cross-sectional study was conducted during 2013/2014 and 2018/2019. The main aim of the analysis was to compare BMI values) and in particular the incidence of underweight, overweight, and obesity between the years2013/2014 and 2018/2019. The *z*-score values, as well as the classification according to BMI, were created based on the World Health Organization (WHO) standards. 

### 2.1. Participants 

The study was conducted in randomly selected primary and secondary schools in Ukraine. Sample sizes were determined using the EPI INFO (StatCalc, Atlanta, GA, USA) software. 

The first study was carried out in 2013/2014 and the methodology has been published in detail [13]. In 2013/2014 approximately 25,000 children were selected from 50 elementary and secondary schools in Ukraine. All students from the selected schools were invited to participate in the study, and 15,456 parental approvals were received for the participation of their children in the study. Inclusion criteria were: obtaining informed consent from each participant and their parents or guardians, being enrolled in the selected schools, a functional state that allows for maintaining a standing position independently, not taking medication affecting body weight, and ages between 7 and 17 years old. Exclusion criteria were: a functional state that does not allow for self-maintenance of a standing position, taking medication affecting body mass, aged less than 7 years or greater than 17 years, a lack of desire to participate in the study or strong pre-test anxiety, and being absent from school on assessment days. Out of 15,456 students whose parents gave approvals for examination, 2009 students were excluded from the study for different reasons. Ultimately, the study group consisted of 13,447 children and adolescents aged 7–17 years. 

The second study was carried out in 2018/2019 and the methodology has been published in detail [14]. The study was repeated in 2018/2019 in the same schools as in 2013/2014, in accordance with the previous protocol. In 2018/2019, approximately 30,000 children from 50 schools were invited. All students from the selected schools were invited to participate in the study, and 19,745 parental approvals were received for the participation of their children in the study. The inclusion and exclusion criteria were the same as in 2013/2014. Out of 19,745 students whose parents gave approval for the examination, 1601 students were excluded because they did not meet the inclusion criteria or were absent from school on the day of the examination. Ultimately, the study group consisted of 18,144 children and adolescents aged 7–17 years. 

The child’s exact age was calculated from the difference between the date of the examination and the date of birth. Exact ages were classified into age groups x (where x = 7 up to 17) by the placement of exact age within the interval (x − 0.5 years, x + 0.5 years). 

### 2.2. Anthropometric Measurements

The examinations were carried out in the offices of school nurses in the morning. Assessments were performed by the same group of researchers. Height and weight were measured for each participant. These measurements were made with the students in their underwear and without shoes. Each measurement was taken as the mean of three consecutive measurements. Body mass was measured with the medical scale RADWAG WPT 60/150 (RADWAG, Radom, Poland) with an accuracy of ±100 g, and height of the children with a stadiometer attached to the scales with an accuracy of ±0.1 cm. All scales were tared using a standardized 1 kg weight. 

Mean values of height and weight were obtained from three measurements in order to calculate body mass index (BMI) according to the formula: BMI = body mass in kg/height in m^2^. Childhood underweight, overweight, and obesity in our study were defined according to the World Health Organization (WHO) child growth standard. Based on the WHO Reference 2007, children aged 6–19 years are overweight and obese with excess weight over 1 SD and 2 SD, respectively, and underweight under 2 SD [15].

### 2.3. Statistical Analysis

Prevalence ratios were calculated with a 95% confidence interval (c.i.). The *z*-scores for the studied children were determined. The *z*-scores were constructed based on the Box-Cox transformation (recommended by the WHO) in such a way that the value of 0 corresponds to the median of the standard BMI distribution for a given age and sex. During calculations, BMI distributions for particular age groups and sex were taken into account. Statistical analysis was performed using the STATISTICA 13.0 software (StatSoft, Inc., Tulsa, OK, USA). Statistical significance and difference was denoted with *, **, or *** (for *p* ≤ 0.05; *p* ≤ 0.01 and *p* ≤ 0.001 respectively). Differences between studies were assessed using the Mann–Whitney test (for *z*-score) and chi-square test. In addition, the relative risk of incidence of overweight, obesity, and underweight was calculated for the 2018/2019 vs. 2013/2014 study.

### 2.4. Ethics

Written informed consent was obtained from parents and children prior to participation in the study. The study was conducted in accordance with the ethical standards stated in the Declaration of Helsinki. The study was approved by Ethics Commission at the Lviv National Medical University, no 4, 17 December 2013 and Bioethics Committee of the Medical Department of the University of Rzeszów, decision no 2015/12/15 on 2 December 2015.

## 3. Results

The analysis covers 31,591 children from Ukraine at two time points. The study of somatic development was conducted in 2013/2014—13,447 children, and 2018/2019—18,144 children. The exact age and sex distribution of the children in both studies are presented in Table 1.

**Figure 1:**
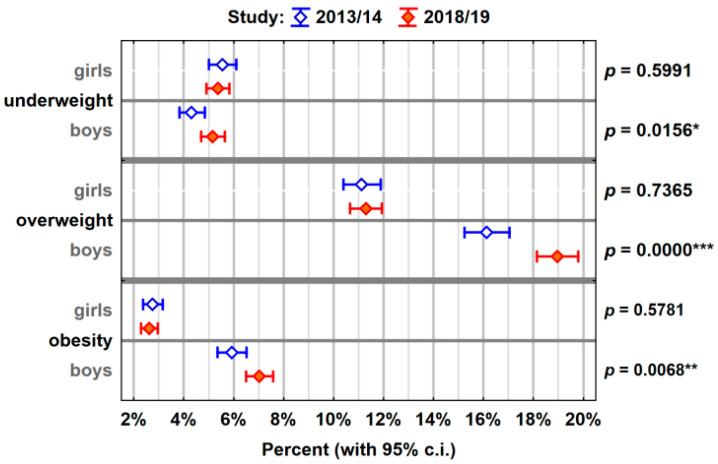
A comparison between the prevalence of underweight, overweight, or obesity in both studies among girls and boys. (*p* < 0.05 (*), *p* < 0.01 (**), *p* < 0.001 (***)).

Figure 1 shows the percentage of children (with a 95% confidence interval) with abnormal body weight (underweight, overweight, or obese) in 2013/2014 and in 2018/2019. The compilation was prepared taking into account the gender division of children. There are statistically significant differences in the percentage of boys who are underweight (*p* = 0.0156 *), overweight (*p* = 0.0000 ***), and obese (*p* = 0.0068 **) between the study from 2013/2014 and the study from 2018/2019. In the later study, there is a greater proportion of boys who are underweight, more are overweight and more obese. However, there are no statistically significant differences between the two studies in the percentage of girls who are underweight, overweight, or obese.

Table 2 shows the mean values, medians, and standard deviation for the *z*-score of girls and boys in terms of age and study period. The value of the Mann–Whitney test assessing the significance of differences in the *z*-score distribution for individual age groups between children from the 2013/2014 and 2018/2019 study is given. It was observed that in 2018/2019, the *z*-score values were significantly lower for most age groups of girls. Exceptions were the 11, 12, 14, and 15-year-olds, where no significant differences were found. In the group of 13-year-old girls, the *z*-score values were significantly lower in the 2013/2014 study. Table 2 also shows the mean values, medians, and standard deviation for the *z*-score of boys by age and study. The value of the Mann-Whitney test assessing the significance of differences in the *z*-score distribution for individual age groups between the boys from the 2013/2014 and 2018/2019 study is given. In the youngest age bands, lower *z*-scores were observed in the 2018/2019 study in the group of 12, 14, and 15-year-olds, significantly higher *z*-scores were found in the 2018/2019 study.

Table 3 shows the relative risk (RR) of overweight/obesity in 2018/2019 compared to 2013/2014. In the group of girls, when considering individual age groups, a significant difference between the study of 2013/2014 and 2018/2019 was observed only among 7-year-olds. The percentage of girls at this age exceeding the BMI norm was lower in the 2018/2019 study. Among boys, a significant difference also applies to 7-year-olds, and, as in girls, a lower share of overweight and obesity was observed in 2018/2019. The significant differences for the ages of 12, 13, and 15 were of a different nature—more overweight or obese boys were found in the 2018/2019 study. 

The share of underweight children was at a similar level for the majority of age groups in both sexes and did not differ in a statistically significant way (Table 4). The exception was a higher incidence of underweight in 2018/2019 among boys aged 7 and 9, and a lower incidence of underweight in 2018/2019 among girls aged 13–14 (Table 4).

## 4. Discussion

Being overweight and obese increases the risk of many serious diseases, therefore screening and monitoring children and adolescents is a very important element of health care. The health risks of overweight and obesity in children include hypertension, hyperinsulinemia, glucose intolerance, type II diabetes, dyslipidemia, increased risk of early cardiac conditions, and interpersonal problems [16]. Long-term monitoring of trends in the prevalence of overweight and obesity among children and adolescents is associated with several difficulties. They may result from the evolving definition of overweight and obesity in children, different age ranges used in studies on the prevalence of overweight and obesity, different methods of selecting the study group, and the applied statistical methods [17]. This study represents the first comparative information on trends in underweight, overweight, and obesity status in children and adolescents in Ukraine. This study indicated a higher incidence of underweight in 2017 among boys aged 7 and 9, and a lower incidence of underweight in 2018/2019 among girls aged 13–14. It can be observed that in children aged 7–12, both in girls and boys, the number of underweight children increased within five years. Similar relations were observed in Greece, where more girls than boys aged 4–17 were thin (8.4% vs. 6.5%). In the entire population, the percentage of underweight children decreased with age in both sexes [18]. The studies indicate that a higher prevalence of underweight was observed in girls than in boys [3].

In the group of girls, a significant difference between the 2013/2014 and 2018/2019 study is observed only among 7-year-olds. The percentage of girls at this age exceeding the BMI norm was lower in the 2018/2019 study. A similar relationship was found among boys and also in 7-year-olds. As for girls, there is a lower share of overweight and obesity in 2018/2019. However, for boys aged 12, 13, and 15, the significant differences were of a different nature—more overweight or obese boys were found in the 2018/2019 study. These results are worrying since higher BMI is related to higher health care costs as well as shorter life expectancy [19,20]. Studies show that overweight and obesity are more common in boys than in girls. However, Mladenova and Andreenko, in examining Bulgarian children, arrived at opposing results [21]. Over the past decade, an increase in the prevalence of overweight and obesity has been noted among Bulgarian children and adolescents. A relatively high percentage of underweight children is observed especially in the group of girls. Overweight or general obesity is more common in boys than in girls [21]. An inverse relationship among 7-year-olds was observed in Hungary [22]. The prevalence of overweight and obesity in Hungarian children (aged 7.0–7.9 years) according to various classifications in 2010–2016 indicated the possible stability of the prevalence of overweight and obesity [22]. Our results indicated an unfavorable increase in the incidence of overweight and obesity with age in boys. Similar trends were observed among boys in Cyprus [23]. However, the studies found that the rates of increase in overall childhood obesity were the same for boys and girls in all age groups in most countries between 1980 and 2015 [24]. In the years 1998–2008, the stabilization of the prevalence of overweight and obesity was observed in school-age children from south-eastern Poland. As in our study, Mazur et al. observed a decreasing incidence of obesity in girls and an increased incidence of overweight in boys [25]. A meta-analysis on the prevalence of overweight and obesity among Greek children showed an increasing trend in this phenomenon (2001–2003), followed by a stabilization (2003–2010) [26]. There has been no increase in the prevalence of overweight/obesity among Portuguese children since 2000 [27]. On the other hand, studies from the north of Poland indicated the incidence of overweight and obesity in total is higher in girls aged 6–7 [28]. Salanve et al. observed that the rates of overweight and obesity among French children did not change significantly between 2000–2007 in children aged 7–9 [29]. G Lazzeri et al. found that the tendency to overweight and obesity among girls aged 9 to 15 decreased sharply in Tuscany, while the incidence of underweight increased [30]. Similar results of overweight and obesity prevalence have been observed in Lithuania [31]. The overweight and obesity among Lithuanian children and adolescents aged 7–17 were more prevalent at a younger age [31]. A study of school children in Turkey showed a significant increase in the prevalence of overweight in 2005–2014 [32]. Increasing tendencies of overweight and obesity in Turkey were confirmed by studies by Celmeli et al. [33]. 

The trend of an increase in overweight and obesity in both sexes, especially in younger children, was also observed in Iranian studies [34]. A 30-year follow-up among Slovenian youth shows a dramatic increase in overweight and obesity over the past three decades. Over the past decade, however, this growth has been reversed or at least stopped. This trend reversal was more pronounced in boys than in girls, and in young children compared to adolescents [35]. At the same time, the prevalence of overweight and obesity in children is very high, but trends have stabilized in most European countries. There are significant differences between countries regarding the current levels and trends of overweight and obesity. The combined prevalence of overweight and obesity in the Iberian region tended to decline, however in the Mediterranean region it increased. No significant changes were observed in either Atlantic or Central Europe [36].

The phenomenon of an increase in the incidence of overweight and obesity may be the result of new life habits, such as an increase in the consumption of unhealthy food and a decrease in daily energy expenditure. Nevertheless, the high prevalence of overweight and obesity in children and adolescents remains a cause for concern [37]. High BMI plays an important role in the disease burden of cardiovascular disease, diabetes, and all causes of mortality. Assessment of levels and trends in exposure to high BMI and the resulting burden of disease underlines the current priority of primary prevention and public health measures focused on obesity [38].

This study has several strengths. High-quality data were obtained from a large representative population group. The data covered several age groups and the data collection methods were consistent and identical throughout the study period. To date, most studies have only looked at the prevalence of overweight and obesity, but not on changes in BMI distribution over time.

In our study, we cannot derive a cause-and-effect relationship. Without longitudinal data, it is impossible to establish a true relationship between the change in BMI among children and adolescents in Ukraine. Moreover, while BMI is generally accepted as an important indicator of body composition for population-level assessment purposes, there are challenges to using BMI as an indicator of obesity. Limitations associated with the use of BMI include inaccurate body weight classifications due to the high ratio of muscle mass to fat mass, variability over time and rate of rapid growth in adolescents, and sexual maturation, all of which are potentially confounding factors.

Our results, combined with evidence about the health consequences of obesity, show that action is needed to reverse the trend in a select age group of children and adolescents. Activities should target social environments where conditions for healthy eating and physical activity can be created. This information should be carefully considered by parents, school principals and teaching staff, pediatricians, and public health professionals when designing more effective strategies to combat the obesity epidemic. Government and stakeholders must develop and implement more effective strategies such as nutrition and physical education programs in schools and public health, and increasing access to and availability of healthy food.

## 5. Conclusions

The percentage of Ukrainian children and adolescents with overweight and obesity increased in 2013–2018 in some age groups, both among boys and girls. Our findings indicated the seriousness of the growing threat posed by overweight and obesity among 7-year-olds in Ukraine. Therefore, any preventive initiatives to stop this trend should focus on the early stages of childhood. At the same time, attention should be paid to the increased incidence of underweight in this age group, especially among 7-year-olds.

## Figures and Tables

**Table 1:** Age and sex distribution of children in both studies.

Age [Years]	Year of the Study			
	2013/2014		2018/2019	
	Boys (*n*)	Girls (*n*)	Boys (*n*)	Girls (*n*)
7	502	533	989	1004
8	619	588	1164	1196
9	639	687	1013	1111
10	656	683	1039	1072
11	613	614	953	1009
12	639	693	874	948
13	629	745	864	911
14	672	747	706	792
15	679	743	629	648
16	465	543	341	476
17	355	403	145	260
Total	6468	6979	8717	9427
	13,447		18,144	

**Table 2:** *Z*-score values with respect to age, gender, and the study—comparison between studies from 2018/2019 and 2013/2014.

Age [yrs.]	Girls							Boys						
	2013/2014			2018/2019				2013/2014			2018/2019			
	Mean	Me	Std. Dev.	Mean	Me	Std. Dev.	*p*	Mean	Me	Std. Dev.	Mean	Me	Std. Dev.	*p*
7	0.19	0.16	1.29	−0.06	−0.05	1.26	0.0006 ***	0.55	0.49	1.50	0.05	0.03	1.49	0.0000 ***
8	0.13	0.18	1.22	−0.03	−0.04	1.24	0.0048 **	0.32	0.30	1.46	0.19	0.17	1.43	0.0589
9	0.06	0.10	1.28	−0.06	−0.02	1.20	0.0442 *	0.40	0.42	1.29	0.24	0.33	1.38	0.0567
10	−0.01	0.01	1.17	−0.13	−0.12	1.22	0.0378 *	0.38	0.41	1.21	0.38	0.47	1.38	0.7839
11	−0.18	−0.13	1.17	−0.24	−0.24	1.15	0.2931	0.25	0.30	1.19	0.20	0.32	1.37	0.8867
12	−0.32	−0.30	1.14	−0.34	−0.24	1.14	0.9999	−0.01	0.04	1.20	0.15	0.19	1.32	0.0052 **
13	−0.45	−0.42	1.11	−0.32	−0.29	1.06	0.0227 *	−0.03	0.06	1.13	0.07	0.14	1.21	0.0569
14	−0.43	−0.38	1.05	−0.34	−0.32	0.96	0.1185	−0.11	−0.03	1.09	0.03	0.07	1.15	0.0180 *
15	−0.41	−0.33	0.97	−0.44	−0.39	0.96	0.4918	−0.14	−0.06	1.02	−0.00	0.01	1.09	0.0130 *
16	−0.41	−0.38	0.84	−0.53	−0.50	0.81	0.0160 *	−0.18	−0.12	0.96	−0.08	−0.07	0.93	0.2096
17	−0.37	−0.44	0.86	−0.55	−0.57	0.84	0.0037 **	−0.09	−0.08	0.93	−0.02	0.01	1.01	0.3518
Total	−0.21	−0.20	1.14	−0.22	−0.21	1.14	0.4192	0.13	0.14	1.22	0.15	0.19	1.32	0.0343 *

*p*-value was calculated using the Mann–Whitney test; *p* < 0.05 (*), *p* < 0.01 (**), *p* < 0.001 (***); yrs.—years; Std. dev.—standard deviation.

**Table 3:** Relative risk of overweight/obesity incidence among Ukrainian children and adolescents—2018/2019 vs. 2013/2014.

Age [yrs.]	Girls						Boys					
	2013/2014		2018/2019		RR (95% C.I.)	*p*	2013/2014		2018/2019		RR (95% C.I.)	*p*
	*N*	%	*N*	%			*N*	%	*N*	%		
7	140	26.3	188	18.7	0.71 (0.59–0.86)	0.0006 ***	190	37.8	252	25.5	0.67 (0.58–0.79)	0.0000 ***
8	139	23.6	250	20.9	0.88 (0.74–1.06)	0.1883	188	30.4	341	29.3	0.96 (0.83–1.12)	0.6358
9	153	22.3	230	20.7	0.93 (0.78–1.11)	0.4299	206	32.2	309	30.5	0.95 (0.82–1.09)	0.4586
10	130	19.0	181	16.9	0.89 (0.72–1.09)	0.2503	196	29.9	349	33.6	1.12 (0.97–1.30)	0.1110
11	93	15.1	150	14.9	0.98 (0.77–1.25)	0.8780	154	25.1	273	28.6	1.14 (0.96–1.35)	0.1264
12	85	12.3	102	10.8	0.88 (0.67–1.15)	0.3430	123	19.2	231	26.4	1.37 (1.13–1.67)	0.0011 **
13	71	9.5	99	10.9	1.14 (0.85–1.52)	0.3725	104	16.5	198	22.9	1.39 (1.12–1.72)	0.0024 **
14	64	8.6	51	6.4	0.75 (0.53–1.07)	0.1125	104	15.5	136	19.3	1.24 (0.99–1.57)	0.0639
15	43	5.8	35	5.4	0.93 (0.60–1.44)	0.7549	80	11.8	112	17.8	1.51 (1.16–1.97)	0.0021 **
16	24	4.4	13	2.7	0.62 (0.32–1.20)	0.1505	42	9.0	42	12.3	1.36 (0.91–2.04)	0.1316
17	26	6.5	11	4.2	0.66 (0.33–1.30)	0.2239	39	11.0	22	15.2	1.38 (0.85–2.24)	0.1943
Total	968	13.9	1310	13.9	1.00 (0.93–1.08)	0.9619	1426	22.0	2265	26.0	1.18 (1.11–1.25)	0.0000 ***

*p*-value was calculated using the chi-square test of independence; *p* < 0.01 (**), *p* < 0.001 (***); yrs.—years.

**Table 4:** Relative risk of underweight among Ukrainian children and adolescents in 2018/2019 vs. 2013/2014.

Age [yrs.]	Girls						Boys					
	2013/2014		2018/2019		RR (95% C.I.)	*p*	2013/2014		2018/2019		RR (95% C.I.)	*p*
	*N*	%	*N*	%			*N*	%	*N*	%		
7	20	3.8	51	5.1	1.35 (0.82–2.25)	0.2380	22	4.4	72	7.3	1.66 (1.04–2.65)	0.0296 *
8	27	4.6	67	5.6	1.22 (0.79–1.89)	0.3693	36	5.8	61	5.2	0.90 (0.60–1.34)	0.6101
9	37	5.4	54	4.9	0.90 (0.60–1.36)	0.6215	25	3.9	68	6.7	1.72 (1.10–2.68)	0.0162 *
10	38	5.6	62	5.8	1.04 (0.70–1.54)	0.8464	21	3.2	50	4.8	1.50 (0.91–2.48)	0.1068
11	39	6.4	66	6.5	1.03 (0.70–1.51)	0.8805	23	3.8	46	4.8	1.29 (0.79–2.10)	0.3118
12	51	7.4	73	7.7	1.05 (0.74–1.48)	0.7962	31	4.9	47	5.4	1.11 (0.71–1.72)	0.6475
13	63	8.5	49	5.4	0.64 (0.44–0.91)	0.0131 *	33	5.2	40	4.6	0.88 (0.56–1.38)	0.5853
14	44	5.9	29	3.7	0.62 (0.39–0.98)	0.0398 *	32	4.8	32	4.5	0.95 (0.59–1.54)	0.8398
15	42	5.7	27	4.2	0.74 (0.46–1.18)	0.2029	31	4.6	21	3.3	0.73 (0.42–1.26)	0.2565
16	18	3.3	21	4.4	1.33 (0.72–2.47)	0.3626	15	3.2	8	2.3	0.73 (0.31–1.70)	0.4586
17	8	2.0	6	2.3	1.16 (0.41–3.31)	0.7779	10	2.8	5	3.4	1.22 (0.43–3.52)	0.7073
Total	387	5.5	505	5.4	0.97 (0.85–1.10)	0.5991	279	4.3	450	5.2	1.20 (1.03–1.38)	0.0156 *

*p*-value was calculated using the chi-square test of independence; *p* < 0.05 (*); yrs.—years.

## Data Availability

The data analyzed during this study are available at the correspondence author e-mail address: kderen@ur.edu.pl.
